# USP4 promotes PTC progression by stabilizing LDHA and activating the MAPK and AKT signaling pathway

**DOI:** 10.18632/aging.206108

**Published:** 2024-10-11

**Authors:** Chuanxiang Hu, Wei Zhang, Yongsheng Jia, Jimin Zhao, Qian Chen, Chengfei Hao, Yang Yu

**Affiliations:** 1 Department of Thyroid and Neck Tumor, Tianjin Medical University Cancer Institute and Hospital, National Clinical Research Center for Cancer, Key Laboratory of Cancer Prevention and Therapy, Tianjin’s Clinical Research Center for Cancer, Tianjin 300060, China; 2 Department of Hepatopancreatobiliary Surgery, Tianjin Nankai Hospital, Tianjin Medical University, Tianjin 300060, China

**Keywords:** USP4, papillary thyroid carcinoma, LDHA

## Abstract

Ubiquitin-specific protease 4 (USP4) has been identified as a promising oncogenic factor implicated in various human malignancies. However, the exact biological functions and underlying mechanisms of USP4 in the progression of papillary thyroid carcinoma (PTC) remain elusive. In this study, we observed a marked upregulation of USP4 expression in PTC tumor tissues. Elevated levels of USP4 were significantly correlated with aggressive clinicopathological features and poor prognosis. Functional assays for loss-of-function demonstrated that silencing USP4 hindered the proliferation of PTC cells. Furthermore, our investigation revealed a specific interaction between USP4 and lactate dehydrogenase A (LDHA), wherein USP4 played a crucial role in stabilizing LDHA protein levels via deubiquitination in PTC cells. Notably, this study demonstrated that USP4 promotes PTC proliferation by modulating the MAPK and AKT signaling pathways. In summary, our findings elucidate the critical involvement of the USP4/LDHA axis in driving PTC progression through the modulation of MAPK and AKT pathways, thereby identifying USP4 as a potential therapeutic target for the treatment of PTC.

## INTRODUCTION

Thyroid cancer represents the most prevalent malignancy within the field of endocrinology. Despite the generally favorable prognosis of most papillary thyroid carcinomas (PTCs), approximately 15% of cases exhibit recurrence with an unfavorable clinical outcome [1]. Unfortunately, the molecular mechanisms underlying the pathogenesis of thyroid cancer remain largely elusive, contributing to a limited understanding of its etiology. Consequently, the therapeutic options for affected individuals remain limited, with only a few treatment modalities currently available.

Ubiquitination represents a critical pathway for protein degradation, meticulously orchestrated by ubiquitylating and deubiquitylating enzymes, collectively termed deubiquitinases (DUBs) [2]. A growing body of evidence underscores the central involvement of ubiquitination and deubiquitination systems in cancer progression, highlighting their potential as therapeutic targets for a spectrum of malignancies. Within the field of DUBs, ubiquitin-specific proteases (USPs) constitute the largest family, characterized by substantial diversity and critical roles in cancer-related biological processes, including cell cycle regulation, DNA damage repair, chromatin remodeling, and modulation of signaling pathways [3–5]. Within the extensive USP family, USP4 holds prominence by orchestrating multiple signaling pathways through its deubiquitination of pivotal proteins [6]. Notably, investigations have highlighted USP4’s elevated expression in colon cancer, where it promotes cell migration by deubiquitinating β-catenin [7]. Similarly, in esophageal squamous cell carcinoma (ESCC), USP4 exhibits significant upregulation, driving ESCC progression by targeting TAK1 [8]. In contrast, the scenario in breast cancer is distinct, with researchers observing a significant downregulation of USP4 expression relative to paired normal breast tissue samples. In breast cancer, USP4 adopts a tumor-suppressive role, inhibiting cell growth through the upregulation of PDCD4 [9]. Noticeably absent from the current literature is a thorough understanding of the biological roles and precise mechanisms underpinning USP4 in thyroid cancer progression. The intricate interplay between USP4 and thyroid cancer remains relatively unexplored, warranting focused investigations to elucidate its contributions to the pathogenesis of this specific malignancy.

The Warburg effect is characterized by the preference of tumor cells to utilize aerobic glycolysis for ATP production, despite the sufficiency of oxygen. The lactate dehydrogenase (LDH) protein family comprises multiple isoforms, including LDHA, LDHB, and LDHC. Among these, LDHA catalyzes the terminal step of the Warburg effect, converting pyruvate and NADH into lactate [10]. Elevated levels of LDHA have been documented in various types of tumors, including papillary thyroid carcinoma (PTC), with higher expression consistently correlating with poorer prognosis [11]. Evidence has demonstrated that LDHA is subjected to the ubiquitin-proteasome pathway. However, the underlying molecular mechanisms governing the ubiquitin-mediated degradation of LDHA in PTC remain elusive.

Although prior studies have elucidated the oncogenic roles of USP4 in various cancers, its function and molecular mechanisms in PTC remain largely unexplored. In this study, we performed additional validations confirming the upregulation of USP4 in patients with PTC and established its correlation with poor prognosis. Furthermore, our findings elucidated a novel mechanism by which USP4 enhances the stability of LDHA through deubiquitination. This process, in turn, activates both the MAPK and AKT signaling pathways, thereby promoting the progression of PTC. Collectively, these results strongly suggest that USP4 exerts pro-oncogenic effects in the context of PTC and may represent a promising therapeutic target for the treatment of this thyroid malignancy.

## MATERIALS AND METHODS

### Bioinformatics analysis

Gene expression profiling was performed on papillary thyroid carcinoma (PTC) tissues and their respective paired normal tissues utilizing data from four GEO databases (GSE3467, GSE6004, GSE33630, and GSE76039). The mRNA levels of key members of the ubiquitin-specific protease (USP) family were subjected to comprehensive analysis.

In parallel, the expression data for USP4 in 505 PTC tissues and 59 normal tissues were extracted from The Cancer Genome Atlas (TCGA) database. Simultaneously, pertinent clinical information associated with these samples was retrieved from the TCGA database. Kaplan-Meier survival plots pertaining to USP4 were generated to assess overall survival based on the available data.

### Cell culture, transfection, and lentiviral infection

This study incorporated four thyroid cancer cell lines (B-CPAP, TPC-1, K1 and KTC-1) along with the HEK293T cell line. All cell lines were cultivated in RPMI-1640 medium or DMEM (Gibco, USA), supplemented with 10% fetal bovine serum (FBS) and 1% penicillin-streptomycin, and maintained under humidified conditions with 5% CO_2_ at 37°C. Lentiviral particles were generated in HEK293T cells through transient co-transfection of transfer vector constructs (pLKO.1-Puro vectors or pCDH-Puro vectors), VSVg, and Delta 8.9. The transfection of HEK293T cells was executed using Lipofectamine 2000 (Invitrogen, USA) following the manufacturer’s protocol.

### Immunohistochemistry (IHC)

The tissue samples of the tumors were subjected to immunohistochemical staining for USP4 according to a standard protocol. The signal was visualized with the DAB Substrate Kit (MaiXin Bio, China). IHC images were visualized using an OLYMPUS BX51 microscope (Olympus, Japan) at 20X and 40X magnification.

### Clinical data and tissue samples

Tissue microarrays including samples from 151 patients with complete medical record information were used in this research. All of the patients were diagnosed with PTC at Tianjin Medical University Cancer Institute and Hospital from January 2013 to June 2014. The research was performed with the approval of the Ethics Committee of the Tianjin Medical University Cancer Institute and Hospital (Ek2023160). Informed consent was obtained for experimentation with human subjects.

### Cell viability and colony formation assay

A seeding density of 1000–1500 cells per well was employed for 96-well plates. Subsequently, cell viability was evaluated using the Cell Counting Kit-8 (UElandy, China) in accordance with the prescribed protocol. For colony formation assays, 500–1000 cells per well were seeded in six-well plates and incubated for 1–2 weeks. The resulting colonies were fixed using 100% methanol for 20 minutes and subsequently stained with 0.5% crystal violet for 15 minutes.

### Reagents, drugs, and antibodies

The following primary antibodies were used in the research: anti-LDHA, anti-phospho-ERK, anti-ERK, anti-phospho-AKT, anti-AKT, anti-GAPDH (Cell Signaling Technology, USA), Anti-USP4 (Santa Cruz Biotechnology, USA). MG132, U0126, LY294002, and SC79 were purchased from Selleck Chemicals (USA). C16-PAF was purchased from MCE (USA).

### Western blotting

Cell proteins were extracted using radioimmunoprecipitation assay buffer (Solarbio, China) following the recommended protocol. The protein sample concentrations were determined using the BCA assay. Subsequently, the samples were subjected to electrophoresis on 8–12% sodium dodecyl sulfate polyacrylamide gel electrophoresis gels and transferred onto polyvinylidene difluoride membranes. Following transfer, membranes were incubated with primary antibodies, followed by peroxidase-conjugated anti-mouse or anti-rabbit IgG antibodies [12]. Visualization of the proteins was achieved using ECL plus reagents (Cell Signaling Technology, USA).

### ATP/ADP ratio

The ATP/ADP ratio was quantified using a commercial ATP/ADP kit obtained from Sigma-Aldrich (Germany), following the provided manufacturer’s instructions. In brief, cells were incubated with ATP reagent for 1 minute, and luminescence was recorded for the ATP assay. Subsequently, after an additional 10-minute incubation, luminescence was measured as the background prior to ADP measurement. Finally, ADP reagent was introduced, and following a 1-minute incubation, luminescence was measured to calculate the ATP/ADP ratio in accordance with the manufacturer’s guidelines.

### Total RNA extraction and quantitative real-time PCR

Total RNA was extracted using TRIzol reagent (Invitrogen, USA), and cDNA was synthesized through reverse transcription using the HiScript III RT SuperMix for qPCR kit (+gDNA wiper) (Vazyme, China) [13]. Subsequent quantitative real-time PCR analysis was conducted utilizing the SYBR Premix Ex Taq II kit (Vazyme, China) with specific primers. The primer sequences for USP4 are provided below: F- CGAGGTGTATTTGCTGGAACTGAAG; R- AATGGTGTCTGCCTTGCTGAAATG.

### Co-immunoprecipitation

For co-immunoprecipitation experiments, B-CPAP cells were collected and immunoprecipitated with 4 µg primary antibodies overnight at 4°C. Then the lysates were incubated with 50 µl protein G Dynabeads for 2 h. The precipitants were washed extensively with wash buffer, boiled with SDS loading buffer, and subjected to SDS-PAGE and immunoblotting.

### Data availability

The data are available on request from the corresponding authors.

## RESULTS

### Differential expression analysis of USP genes in PTC patients

**Figure 1:**
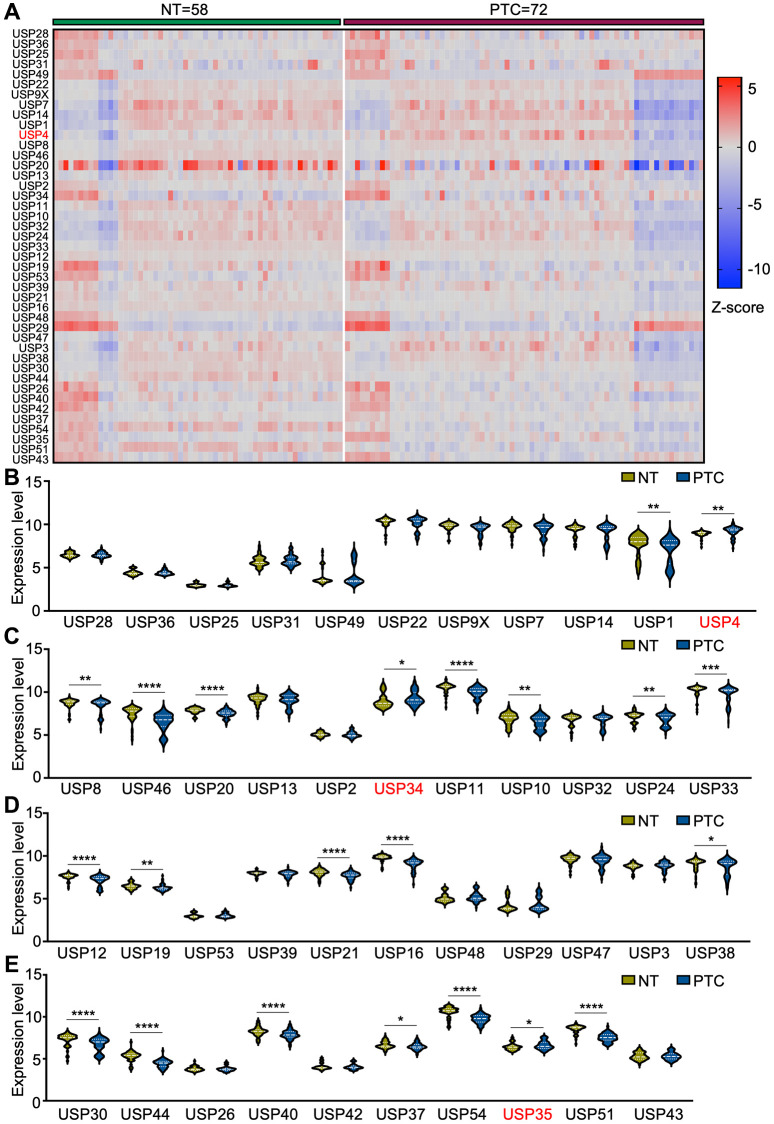
**Differential expression analysis of USP genes in PTC patients.** (**A**) Heat map representing USP gene expression profiles in PTC and adjacent normal thyroid tissues, derived from four GEO databases (GSE3467, GSE6004, GSE33630, and GSE76039); (**B**–**E**) Violin plots illustrating USP gene expression profiles in PTC and adjacent normal thyroid tissues across the four aforementioned GEO databases. All ^*^p < 0.05, ^**^p < 0.01, ^***^p < 0.001, ^****^p < 0.0001.

To elucidate the potential roles of USP genes in PTC tumorigenesis and progression, we analyzed the expression levels of 43 USP genes using four GEO databases (GSE3467, GSE6004, GSE33630, and GSE76039) (Figure 1A). In total, 58 normal thyroid tissues and 72 PTC tissues were included in the analysis. Compared with normal thyroid tissues, 19 genes exhibited significant downregulation in PTC tissues, while USP4, USP34, and USP35 showed significant upregulation (Figure 1B–1E). As one of the key members of the USP family, the function and molecular mechanisms of USP4 in PTC have yet to be elucidated. Therefore, in this study, USP4 was selected as the focus of our investigation.

### Correlations between USP4 expression and clinical features of PTC patients

**Figure 2:**
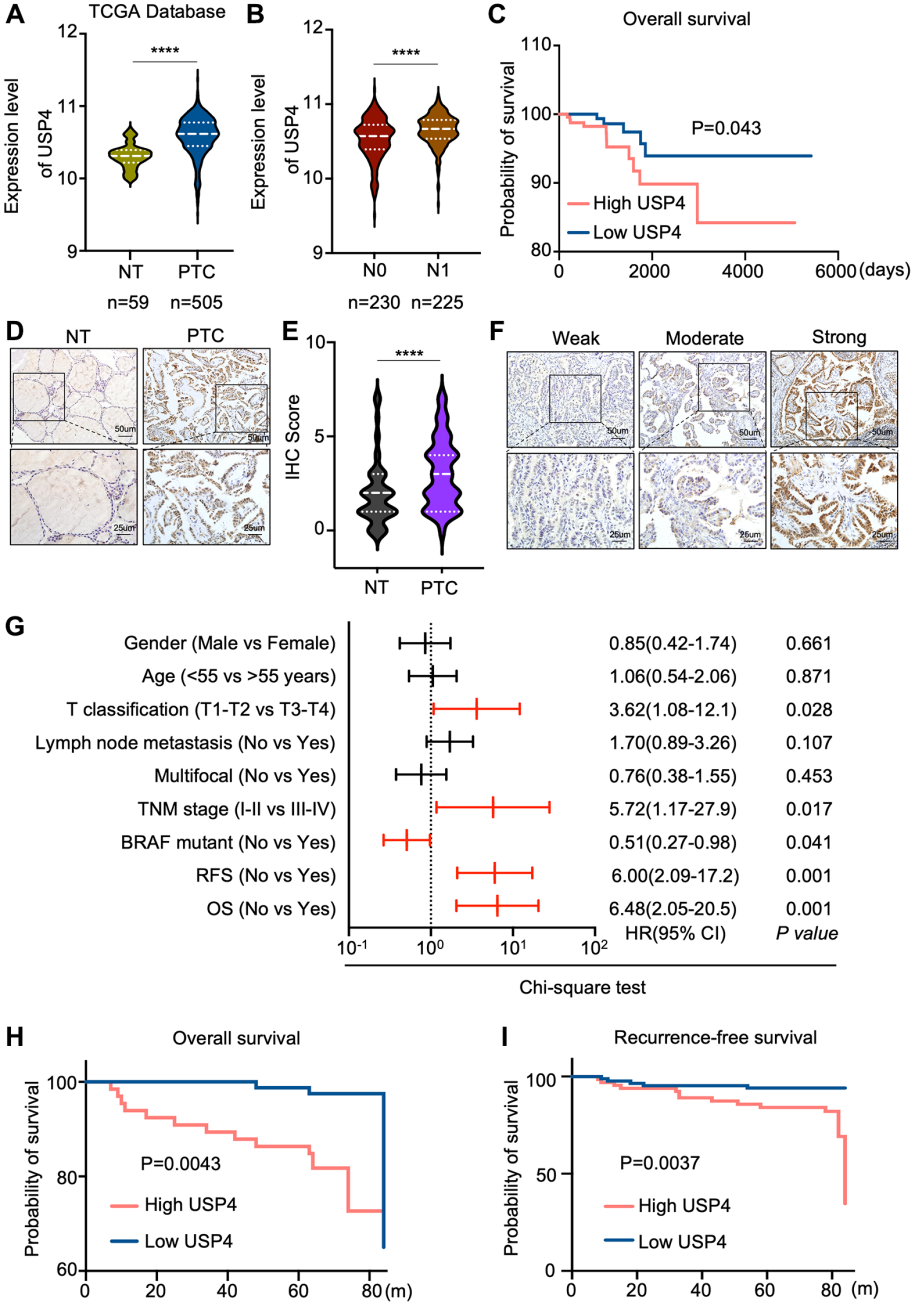
**Correlations between USP4 expression and clinical features of PTC patients.** (**A**) Violin plot depicting USP4 gene expression levels in PTC and adjacent normal thyroid tissues sourced from the TCGA database; (**B**) Comparison of USP4 mRNA expression levels across different N stages in PTC tissues, as sourced from the TCGA database; (**C**) Kaplan-Meier curve estimating overall survival rates of PTC patients from the TCGA database, stratified by varying USP4 expression levels; (**D**) Representative IHC staining images showing USP4 expression in PTCs and their corresponding normal tissues; (**E**) Statistical quantification of IHC scores for USP4 expression in PTCs and their corresponding normal tissues; (**F**) Representative IHC images showing weak, moderate, and strong staining of USP4 in PTCs; (**G**) Univariate analysis examining the associations between USP4 protein levels and clinicopathologic features in PTC patients from the validation cohort. Red lines denote clinicopathologic features significantly associated with USP4 expression. (**H**, **I**) Kaplan-Meier curves for overall survival (OS) and recurrence-free survival (RFS) illustrating PTC patient outcomes in relation to high versus low USP4 expression levels. All ^*^p < 0.05, ^**^p < 0.01, ^***^p < 0.001, ^****^p < 0.0001.

To validate the differential expression of USP4 in PTC tissues, we analyzed its expression in the TCGA database, which includes 505 PTC tissues and 59 normal thyroid tissues. Consistently, our analysis revealed a significant upregulation of USP4 in PTC tissues compared to normal thyroid tissues (Figure 2A). To elucidate the clinical relevance of USP4 in PTC, we examined its correlation with key clinicopathologic parameters in both the validation cohort and the TCGA database. Notably, elevated USP4 levels were associated with advanced N stages, indicating a propensity for disease progression (Figure 2B). Furthermore, heightened USP4 expression consistently correlated with shorter overall survival (OS) (Figure 2C).

To corroborate these findings, we performed immunohistochemical (IHC) analysis on a cohort of 151 PTC patients. The results underscored a significant increase in USP4 expression in PTC tissues compared to corresponding adjacent normal thyroid tissues (Figure 2D, 2E). Moreover, univariate logistic regression analysis based on USP4 expression levels in PTC revealed a strong correlation with T stages, TNM stages, BRAF mutation status, recurrence-free survival, and overall survival (Figure 2F, 2G). Additionally, Kaplan-Meier analysis demonstrated a significant association between higher USP4 expression levels and shorter overall survival (OS) and recurrence-free survival (RFS) in PTC (Figure 2H, 2I). These comprehensive findings further underscore the clinical significance of USP4 as a potential prognostic marker in PTC.

### USP4 promotes the proliferation of PTC

**Figure 3:**
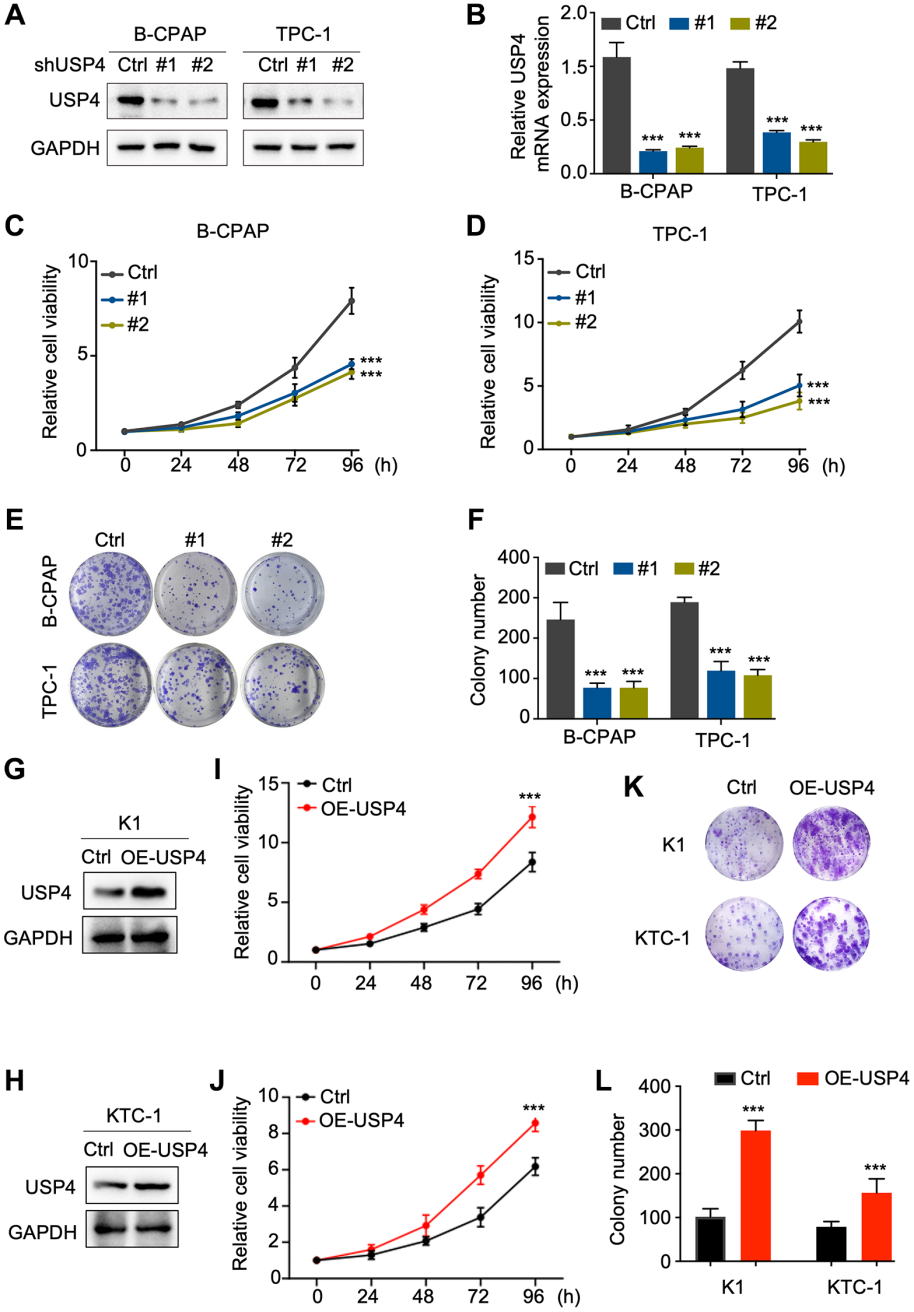
**USP4 promotes the proliferation of PTC.** (**A**) Western blot analysis to determine the knockdown efficiency of USP4 in B-CPAP and TPC-1 cells; (**B**) qPCR analysis to determine the knockdown efficiency of USP4 in B-CPAP and TPC-1 cells; (**C**, **D**) CCK-8 assay to assess the proliferative capacity of B-CPAP and TPC-1 cells following USP4 knockdown; (**E**) Representative images from colony formation assays of B-CPAP and TPC-1 cells with USP4 knockdown; (**F**) Statistical quantification of colony numbers in B-CPAP and TPC-1 cells with USP4 knockdown; (**G**, **H**) Western blot analysis to determine USP4 overexpression in K1 and KTC-1 cells; (**I**, **J**) CCK-8 assay to assess the proliferative capacity of K1 and KTC-1 cells expressing USP4; (**K**) Representative images from colony formation assays of K1 and KTC-1 cells expressing USP4; (**L**) Statistical quantification of colony numbers in K1 and KTC-1 cells expressing USP4. All ^*^p < 0.05, ^**^p < 0.01, ^***^p < 0.001, ^****^p < 0.0001.

To investigate the potential impact of USP4 on the biological behavior of PTC cells, we knocked down USP4 expression in B-CPAP and TPC-1 cells. Western blot analysis and qPCR were performed to assess knockdown efficiency, revealing significant reductions in USP4 protein and mRNA levels (Figure 3A, 3B). Furthermore, the CCK-8 assay demonstrated that USP4 knockdown significantly inhibited the proliferation of both B-CPAP and TPC-1 cells (Figure 3C, 3D). Moreover, the colony formation assay indicated that USP4 knockdown significantly reduced colony number in both B-CPAP and TPC-1 cells (Figure 3E, 3F). Finally, to further elucidate the role of USP4 in PTC, we overexpressed USP4 in K1 and KTC-1 cells (Figure 3G, 3H). CCK-8 and colony formation assays demonstrated that USP4 overexpression promotes PTC cell proliferation (Figure 3I–3L).

### USP4 facilitates PTC progression via the MAPK and AKT pathways

**Figure 4:**
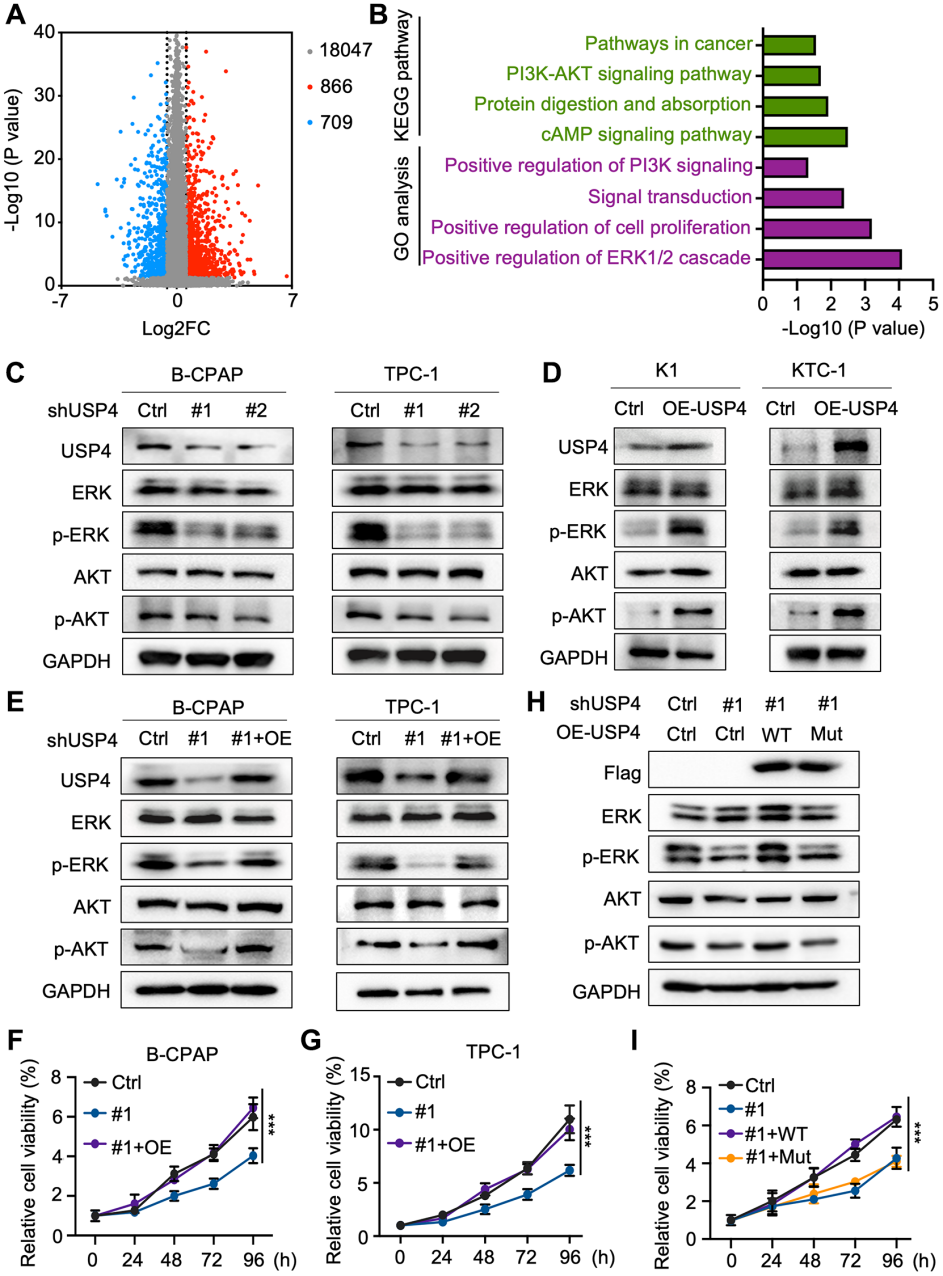
**USP4 facilitates PTC progression via the MAPK and AKT pathways.** (**A**) Volcano plot depicting gene expression changes in tissues with high versus low USP4 expression, generated from TCGA data; (**B**) Summary of significantly enriched pathways in tissues exhibiting high versus low USP4 expression; (**C**) Western blot analysis to evaluate changes in ERK, phosphorylated ERK, AKT, and phosphorylated AKT levels in B-CPAP and TPC-1 cells following USP4 knockdown; (**D**) Western blot analysis to examine changes in ERK, phosphorylated ERK, AKT, and phosphorylated AKT levels in K1 and KTC-1 cells overexpressing USP4; (**E**) Western blot analysis to investigate changes in ERK, phosphorylated ERK, AKT, and phosphorylated AKT levels in B-CPAP and TPC-1 cells after USP4 restoration; (**F**, **G**) CCK-8 assay to evaluate the proliferative capacity of B-CPAP and TPC-1 cells following USP4 knockdown and subsequent restoration; (**H**) Western blot analysis to investigate alterations in ERK, phosphorylated ERK, AKT, and phosphorylated AKT levels in USP4-knockdown cells subsequently expressing either wild-type USP4 or a deubiquitination-deficient mutant (C311A); (**I**) CCK-8 assay to evaluate proliferation in USP4-knockdown cells subsequently expressing either wild-type USP4 or a deubiquitination-deficient mutant (C311A). All ^*^p < 0.05, ^**^p < 0.01, ^***^p < 0.001, ^****^p < 0.0001.

To elucidate the molecular mechanisms by which USP4 influences PTC tumorigenesis, we screened for differentially expressed genes associated with high and low USP4 expression in PTC patients using the TCGA database. The analysis identified 866 significantly upregulated genes and 709 significantly downregulated genes (Figure 4A). Global pathway analysis indicated significant enrichment in the “positive regulation of the ERK1/2 cascade” and the “PI3K-AKT signaling pathway” (Figure 4B). Subsequently, we verified the influence of USP4 on the MAPK and AKT signaling pathways by Western blot analysis. The results demonstrated that USP4 knockdown significantly reduced levels of phosphorylated ERK and phosphorylated AKT, whereas USP4 overexpression significantly elevated these levels (Figure 4C, 4D). Meanwhile, we investigated the functional role of USP4 mediated by the MAPK and AKT signaling pathways. The results indicated that USP4 overexpression in PTC cells, where USP4 was originally knocked down, significantly reversed the inhibition of the MAPK and AKT signaling pathways (Figure 4E). Concurrently, CCK-8 and colony formation assays showed that USP4 overexpression in USP4-knockdown cells significantly rescued USP4-mediated effects on cell proliferation (Figure 4F, 4G and Supplementary Figure 1A, 1B). Furthermore, to investigate the role of USP4’s deubiquitination activity, we engineered USP4-knockdown cells to express either wildtype USP4 or a deubiquitination-deficient mutant (C311A). The results demonstrated that wildtype USP4, but not the C311A mutant, reversed the inhibition of MAPK and AKT signaling pathways (Figure 4H). Additionally, CCK-8 assay revealed that wildtype USP4, in contrast with the C311A mutant, restored PTC proliferation (Figure 4I). In conclusion, USP4 promotes PTC progression through the MAPK and AKT pathways, contingent upon its deubiquitination activity.

### MAPK and AKT pathway inhibitors and agonists reverse the impact of USP4 in PTC

**Figure 5:**
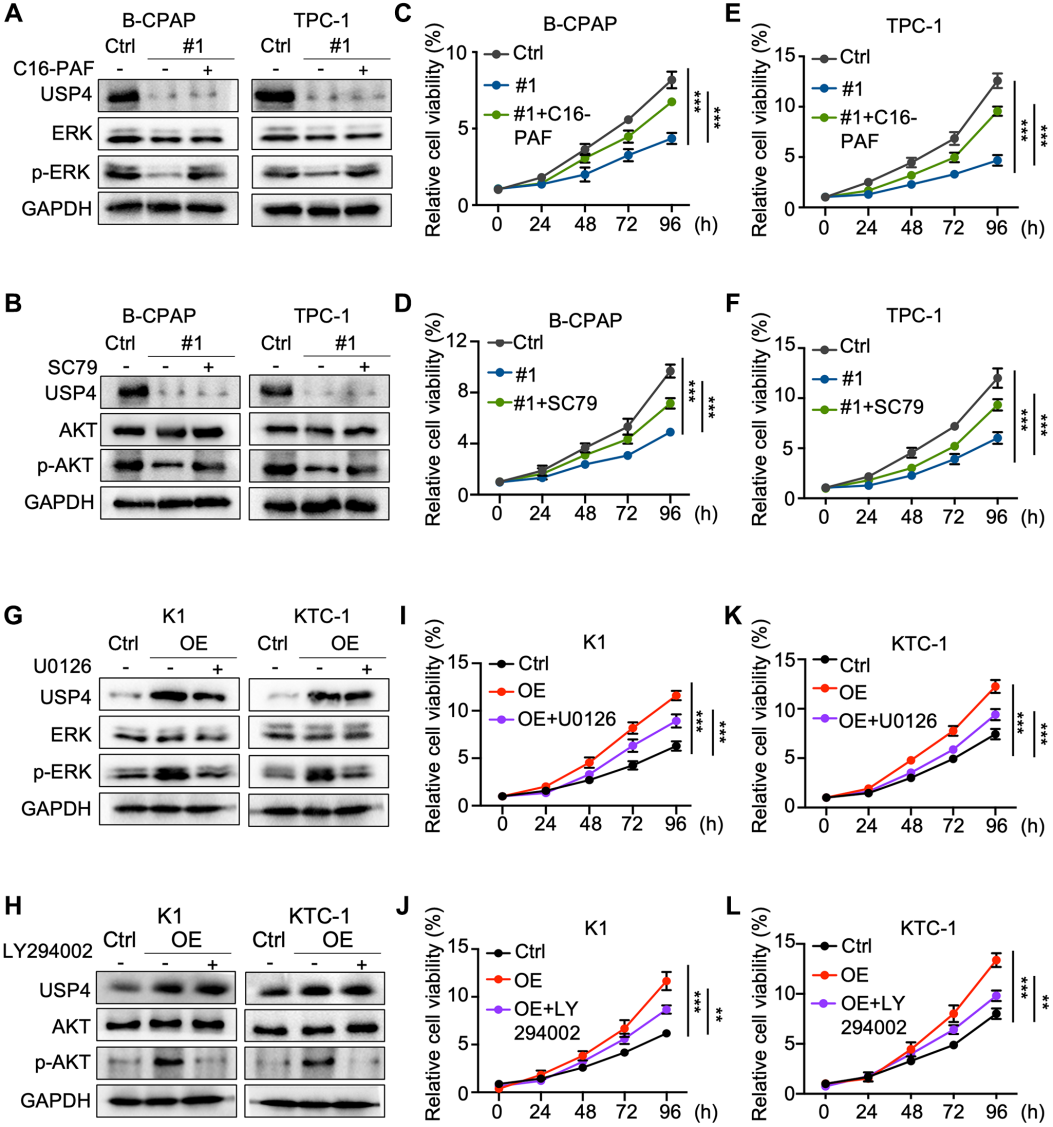
**MAPK and AKT pathway inhibitors and agonists reverse the impact of USP4 in PTC.** (**A**, **B**) Western blot analysis for detecting USP4, ERK, AKT, phosphorylated ERK, and phosphorylated AKT in B-CPAP and TPC-1 cells after USP4 knockdown and subsequent treatment with C16-PAF (10 μM for 24 h) or SC79 (4 μg/mL for 24 h); (**C**–**F**) CCK-8 assay to evaluate the proliferative capacity of B-CPAP and TPC-1 cells after USP4 knockdown and subsequent treatment with C16-PAF (10 μM for 24 h) or SC79 (4 μg/mL for 24 h); (**G**, **H**) Western blot analysis to detect USP4, ERK, AKT, phosphorylated ERK, and phosphorylated AKT in K1 and KTC-1 cells overexpressing USP4 after treatment with U0126 (20 μM for 24 h) or LY294002 (50 μM for 24 h); (**I**–**L**) CCK-8 assay to evaluate the proliferative capacity of K1 and KTC-1 cells overexpressing USP4 after treatment with U0126 (20 μM for 24 h) or LY294002 (50 μM for 24 h). All ^*^p < 0.05, ^**^p < 0.01, ^***^p < 0.001, ^****^p < 0.0001.

To further explore the roles of MAPK and AKT signaling pathways in the molecular regulation through which USP4 modulates PTC cell tumorigenesis. Initially, we activated the MAPK and AKT signaling pathways in USP4-knockdown cells using C16-PAF and SC79, respectively (Figure 5A, 5B). CCK-8 and colony formation assays demonstrated that C16-PAF and SC79 partially reversed the proliferation inhibition induced by USP4 knockdown (Figure 5C–5F and Supplementary Figure 2A–2D). Additionally, we treated USP4-expressing cells with U0126 and LY294002, inhibitors of the MAPK and AKT signaling pathways, respectively (Figure 5G, 5H). CCK-8 and colony formation assays showed that U0126 and LY294002 significantly mitigated the proliferation effects induced by USP4 overexpression (Figure 5I–5L and Supplementary Figure 2E–2H). In summary, these findings indicate that USP4 promotes PTC progression partially through the MAPK and AKT signaling pathways.

### USP4 enhances PTC proliferation by stabilizing LDHA

**Figure 6:**
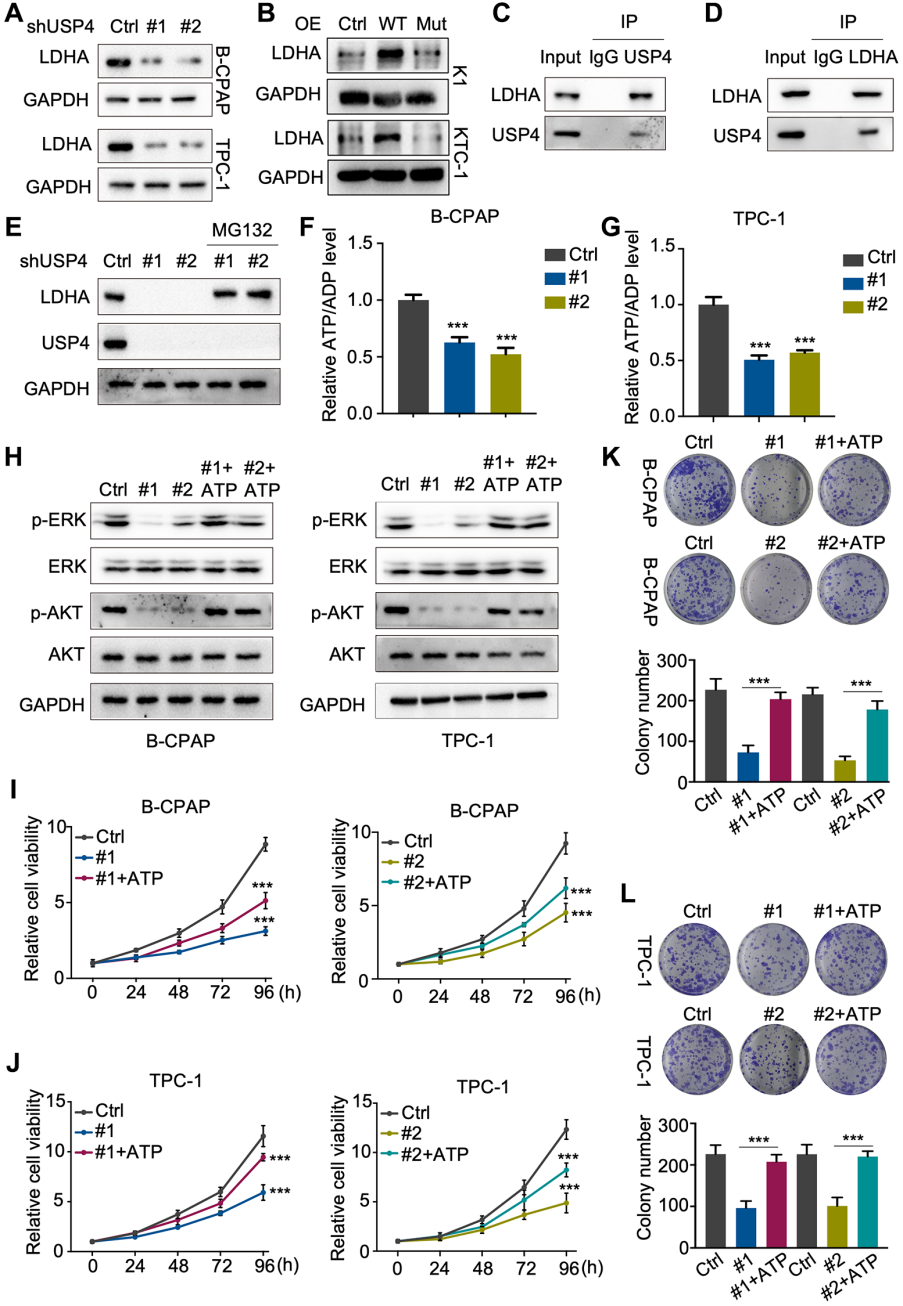
**USP4 enhances PTC proliferation by stabilizing LDHA.** (**A**, **B**) Western blot analysis to detect LDHA following USP4 knockdown in B-CPAP and TPC-1 cells or USP4 overexpression in K1 and KTC-1 cells; (**C**, **D**) B-CPAP cells were lysed with RIPA buffer. The lysates were immunoprecipitated with anti-IgG, anti-USP4, or anti-LDHA antibodies, followed by Western blot analysis with the specified antibodies; (**E**) USP4-knockdown B-CPAP cells were treated with MG132 (10 μM) for 4 hours, followed by Western blot analysis using the specified antibodies to detect proteins; (**F**, **G**) ATP/ADP ratio assay revealed a reduction in the ATP/ADP ratio in B-CPAP and TPC-1 cells following USP4 knockdown; (**H**) Western blot assays were conducted to evaluate changes in ERK, phosphorylated ERK, AKT, and phosphorylated AKT levels in B-CPAP and TPC-1 cells following USP4 knockdown. A similar analysis was performed on B-CPAP and TPC-1 cells with USP4 knockdown subjected to ATP treatment (1 mM); (**I**, **J**) CCK-8 assay was utilized to evaluate the proliferative capacity of B-CPAP and TPC-1 cells following USP4 knockdown and subsequent ATP treatment; (**K**, **L**) Colony formation assays were conducted to investigate the proliferation potential of B-CPAP and TPC-1 cells following USP4 knockdown and subsequent ATP treatment (1 mM). All ^*^p < 0.05, ^**^p < 0.01, ^***^p < 0.001, ^****^p < 0.0001.

In this study, we observed that USP4 knockdown markedly decreased LDHA protein levels in both B-CPAP and TPC-1 cells (Figure 6A). Additionally, the results indicated that wildtype USP4 overexpression, but not the C311A mutant, markedly increased LDHA levels (Figure 6B). Immunoprecipitation (IP) with antibodies against USP4 or LDHA demonstrated that USP4 and LDHA interact with each other (Figure 6C, 6D). Additionally, the proteasome inhibitor MG132 significantly rescued the downregulation of LDHA protein due to USP4 knockdown (Figure 6E). Studies have shown that the absence of LDHA decreases ATP production, leading to the inhibition of PTC. In our study, we found that LDHA knockdown significantly decreased the ATP/ADP levels in B-CPAP and TPC-1 cells (Figure 6F, 6G). Taken together, our findings indicate that USP4 enhances the stability of LDHA, subsequently promoting ATP production in PTC.

ATP has been reported to activate the MAPK and AKT signaling pathways in various systems. Subsequently, we verified the regulatory mechanism of USP4 on the MAPK and AKT signaling pathways using Western blot analysis. The results demonstrated that USP4 knockdown significantly decreased the levels of phosphorylated ERK and phosphorylated AKT, which could be rescued by ATP (Figure 6H). Furthermore, CCK-8 and colony formation assays (Figure 6I–6L) demonstrated that ATP restored the proliferation inhibition due to USP4 knockdown. In summary, our results indicate that USP4 promotes PTC progression through the stabilization of LDHA.

## DISCUSSION

**Figure 7:**
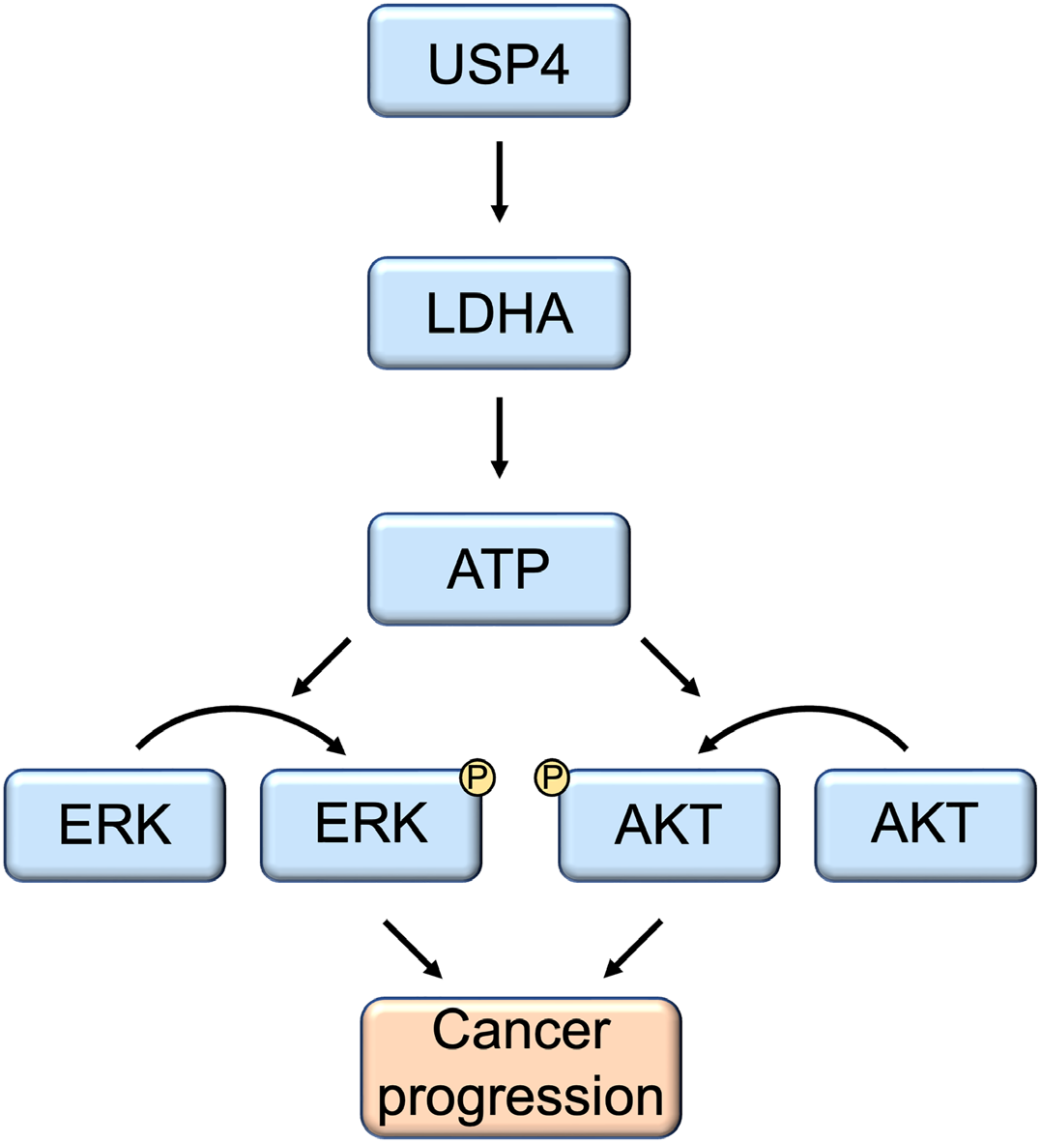
**Schematic model of the function and mechanism of USP4 in thyroid cancers.** USP4 promotes PTC progression by enhancing LDHA protein stability through deubiquitination. Consequently, this process activates both the MAPK and AKT signaling pathways.

Numerous studies have revealed that many members of the USPs consistently predict a poor prognosis in various cancers. Concurrently, certain USPs have been identified as targets for the development of inhibitors in cancer therapy. Here, our research screened the expression of key USP members in PTC and revealed that USP4 is significantly upregulated and consistently indicates a poor prognosis. Further mechanistic studies demonstrated that USP4 promotes PTC progression by enhancing the stability of the LDHA protein through deubiquitination, subsequently activating the MAPK and AKT signaling pathways (Figure 7). These results suggest that USP4 plays a critical role in PTC malignancy, and it may serve as a novel prognostic marker and a promising therapeutic target for PTC.

Mounting evidence supports the assertion that USP4 serves as a promising biomarker and plays a pivotal role in diverse cellular and biological processes associated with various cancers [7, 14–17]. To date, USP4 has demonstrated its potential as a prognostic biomarker across a spectrum of malignancies, including pancreatic cancer, multiple myeloma, and lung cancer [18–21]. In the context of the current investigation, our findings indicate a significant upregulation of USP4 expression in PTC. Additionally, we observed a positive correlation between USP4 expression levels and critical clinical parameters, including tumor size, TNM stage, BRAF mutation, decreased recurrence-free survival time, and decreased overall survival time. Simultaneously, accumulating evidence from both *in vitro* and *in vivo* studies suggests that elevated USP4 expression enhances the proliferative and migratory capabilities of cancer cells [22–24]. Consistent with this, our study elucidates that USP4 depletion markedly impedes PTC proliferation. Consequently, USP4 emerges as a novel prognostic biomarker and a promising therapeutic target for PTC. However, it is noteworthy that divergent roles of USP4 have been identified in other cancer types, such as head and neck squamous cell carcinoma (HNSCC) and breast cancer, where USP4 acts as a tumor suppressor [9, 25]. TNF-α is implicated in apoptosis via the regulation of LDH and mediates cell proliferation and energy metabolism through interaction with its receptor [26, 27]. Notably, USP4 has been shown to negatively regulate RIP1-mediated NF-κB activation and facilitate TNF-α-induced apoptosis in HNSCC. The observed dual role of USP4 in cancer underscores its intricate involvement in diverse cellular pathways, which may be contingent upon cell type-specific variations. This complexity suggests that the impact of USP4 on cancer pathogenesis is nuanced and dependent upon the intricate interplay within distinct cellular contexts.

Further investigation is imperative to elucidate the precise molecular mechanisms underlying the tumor-promoting functions of USP4 in PTC. As a member of the USP family, USP4 regulates multiple signaling pathways by deubiquitinating key proteins [28–30]. Prior research has established that USP4 promotes TGF-β signaling and synergizes with AKT signaling to enhance the invasion and migration of cancer cells [31, 32]. Lactate dehydrogenase A (LDHA) serves as a pivotal enzyme catalyzing the terminal step of the Warburg effect, converting pyruvate and NADH to lactate, thus playing a crucial role in tumor metabolism and growth [11]. Emerging evidence indicates that LDHA is degraded via the ubiquitin-proteasome pathway [33]. Our findings reveal that USP4 deubiquitinates and stabilizes LDHA, thereby promoting ATP production. It is well-established that extracellular ATP can activate the PI3K/AKT and MAPK signaling pathways in specific cell types [34–36]. In our study, we demonstrate that USP4 activates the AKT and MAPK signaling pathways by augmenting ATP production, consequently promoting PTC progression. Nonetheless, a more intricate understanding of the molecular intricacies governing this phenomenon and the clinical implications of this pathway in PTC necessitates further exploration in future studies.

Collectively, USP4 expression is significantly elevated in PTC, consistently correlating with a poor prognosis. Furthermore, USP4 regulates the protein levels of lactate dehydrogenase A (LDHA) via the ubiquitin-proteasome system, thereby contributing to PTC progression. This underscores the potential of USP4 as a molecular target for therapeutic interventions in PTC treatment.

## Supplementary Materials

Supplementary Figures
