# Supplementary material for: USP4 promotes PTC progression by stabilizing LDHA and activating the MAPK and AKT signaling pathway
Source: Aging (Albany NY). 2024 Oct 11;16(19):12850–65. doi: 10.18632/aging.206108 (PMC11501377; doi:10.18632/aging.206108)
Supplement: Supplementary Figures [file aging-16-206108-s001.pdf]

SUPPLEMENTARY FIGURES

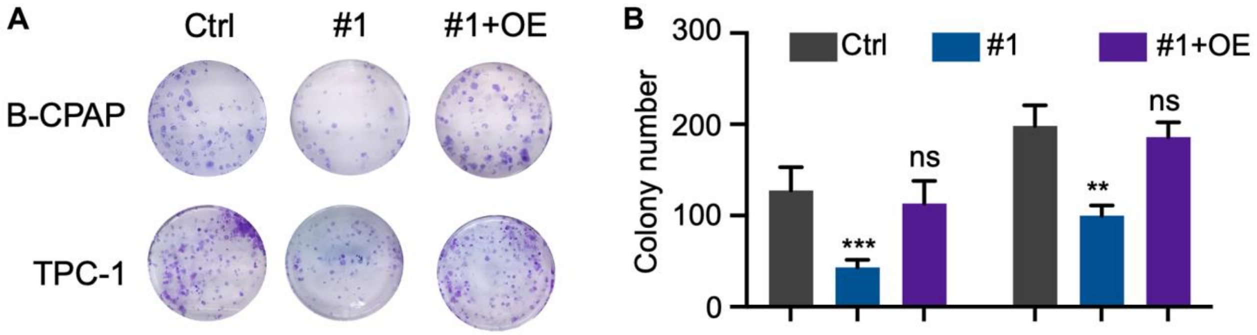

Supplementary Figure 1. USP4 facilitates PTC progression via the MAPK and AKT pathways. (**A**) Representative images from colony formation assays of B-CPAP and TPC-1 cells post USP4 knockdown and reconstruction; (**B**) Statistical quantification of colony formation in B-CPAP and TPC-1 cells post USP4 knockdown and reconstruction. All $^{*}p < 0.05$, $^{**}p < 0.01$, $^{***}p < 0.001$, $^{****}p < 0.0001$.

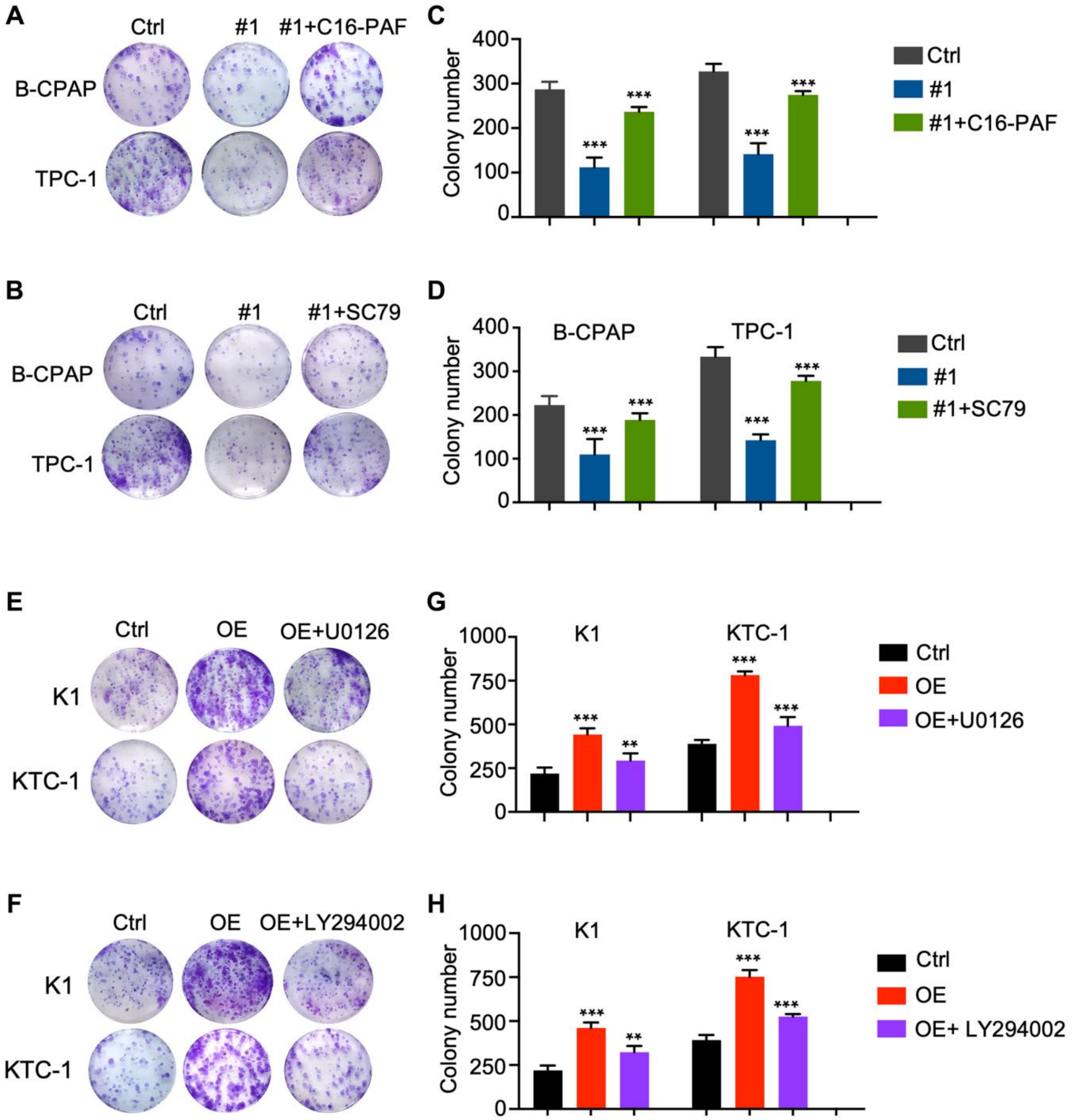

Supplementary Figure 2. MAPK and AKT pathway inhibitors and agonists reverse the impact of USP4 in PTC. (**A**, **B**) Representative images from colony formation assays of B-CPAP and TPC-1 cells post USP4 knockdown following C16-PAF (10 μM for 24 h) or SC79 (4 μg/mL for 24 h) treatment; (**C**, **D**) Statistical quantification of colony formation in B-CPAP and TPC-1 cells post USP4 knockdown following C16-PAF or SC79 treatment; (**E**, **F**) Representative images from colony formation assays of K1 and KTC-1 cells expressing USP4 following U0126 (20 μM for 24 h) or LY294002 (50 μM for 24 h) treatment; (**G**, **H**) Statistical quantification of colony formation in K1 and KTC-1 cells expressing USP4 following U0126 or LY294002 treatment. All $^{*}p < 0.05$, $^{**}p < 0.01$, $^{***}p < 0.001$, $^{****}p < 0.0001$.
